# Price Comparisons on the Internet Based on Computational Intelligence

**DOI:** 10.1371/journal.pone.0106946

**Published:** 2014-09-30

**Authors:** Jun Woo Kim, Sung Ho Ha

**Affiliations:** 1 Department of Industrial & Management Systems Engineering, Dong-A University, Busan, Korea; 2 School of Business Administration, Kyungpook National University, Daegu, Korea; Politehnica University of Bucharest, Romania

## Abstract

Information-intensive Web services such as price comparison sites have recently been gaining popularity. However, most users including novice shoppers have difficulty in browsing such sites because of the massive amount of information gathered and the uncertainty surrounding Web environments. Even conventional price comparison sites face various problems, which suggests the necessity of a new approach to address these problems. Therefore, for this study, an intelligent product search system was developed that enables price comparisons for online shoppers in a more effective manner. In particular, the developed system adopts linguistic price ratings based on fuzzy logic to accommodate user-defined price ranges, and personalizes product recommendations based on linguistic product clusters, which help online shoppers find desired items in a convenient manner.

## Introduction

The Internet is a fundamental infrastructure that integrates distributed and heterogeneous networks, communication, and information systems to provide information-convergent computing environments [1]. In addition, new communication technologies have changed the manner in which individuals access and acquire information from various information sources [2]. Many Web sites and Web services are based on the flux of information convergence and Web users enjoy a wide access to abundant information from various sources through consolidated channels, services, and Web sites, among other means [3].

However, end users may have some difficulty in combining, transforming, and processing massive amounts of gathered information, which may result in irrelevant search results, fraudulent transactions, and dispersed information [4]–[5]. Therefore, many users may become disoriented and face worsening problems of information overload and uncertainty when browsing information-intensive Web sites [6].

A good example is a price comparison site (PCS), (also known as shopbots or comparative shopping agents), providing online shoppers with opportunities to acquire a wide range of information on various products. It is well known that a PCS can help online shoppers reduce the amount of time or effort required when searching for products online [7]–[10]. However, such sites are generally designed to focus mainly on the needs of “expert” shoppers. Therefore, many users tend to be overwhelmed by the enormous amount of information on a plethora of products from various vendors [11]. In addition, there are two major approaches to information-seeking through the Web, i.e., direct searching and browsing [12]. Conventional PCSs are generally suitable for direct searches, which focus on locating the required information on specific products, but do not effectively support browsing, which focuses on finding “something useful.”

For this study, an intelligent product search system was developed that enables PCSs to support novice shoppers specifically by accommodating user-defined price ranges. Herein, a “novice shopper” is defined as an online shopper who is interested in a certain product category and wishes to make a purchase within an approximate budget, but who is having difficulty selecting a specific product owing to a lack of prior knowledge on the target product category.

For this study, linguistic price ratings and linguistic product clusters were therefore devised that employ a linguistic-semantic extraction technique such as fuzzy logic [5] [13]–[14] and data mining [15], which have emerged as useful tools for processing information collected from Web sites and providing personalized Web services [4] [6]. In addition, the present study provides important insight into various problems embedded in information-intensive Web sites such as PCSs, and suggests some service strategies for addressing these problems.

The rest of this paper is organized as follows. Section 2 provides a review of previous research on PCSs. Section 3 explains the limitations of existing PCSs and describes the overall framework of the proposed intelligent product search system. Section 4 provides the experimental results obtained by applying the proposed system to a popular PCS in Korea. Finally, Section 5 provides some concluding remarks and discusses some interesting avenues for future research.

## Literature Review

As online shopping increases in popularity, PCSs have become one of the most important Web-based business intermediaries for both merchants and online shoppers [16]. Typically, comparison sites gather information on products and their prices imposed by different merchants, and enable online shoppers to select products and merchants to make purchase decisions in effective manners [17]. It is well known that such Web sites can dramatically reduce the search cost during online shopping [16], which has led many online shoppers to begin their purchasing procedure by visiting a PCS such as Nextag.com, PriceGrabber.com, or Bizrate.com [18]–[20].

Owing to their widespread use, PCSs have attracted a great deal of attention from researchers and practitioners. Typically, the role of a PCS is to locate the best merchant quoting the lowest price for a specific product. In this context, many previous researchers have approached the use of PCSs from social and economic perspectives, and the price dispersion has been a major topic of research [21]. Indeed, many studies have suggested that the low search cost of a PCS can facilitate the convergence of prices for identical products [8]. However, the price dispersion still remains, and some studies have reported that the extent of such price dispersion may be influenced by various factors such as the product category, number of sellers, and market imperfections [7] [22]–[24]. Similarly, user behaviors and industrial influences on PCSs in providing accessibility to the lowest prices have also been discussed and actively studied [25]–[28].

In addition, owing to the large number of similar products and merchants on the Web, online shoppers may feel disoriented when facing the massive amount of information provided by a PCS [29]–[31]. Indeed, conventional price-comparison agents help in determining “where to buy” a specific product; however, they do not appropriately support individual shoppers in determining “what to buy.” That is, it is generally assumed that online shoppers visit a PCS after determining to purchase a specific product [16]. However, it is well known that the shopping process generally starts with the “what to buy” phase, where the shoppers determine specific products suitable for their customized needs [32]. Therefore, traditional filtering and order-based PCSs are insufficient, and a more comprehensive and intelligent purchase-decision support is required [5] [33]–[34].

There have been several studies dealing with purchase-decision support of PCSs. Yuan [22] argued that prices are insufficient for finding recommendable products, and proposed an intelligent comparison-shopping agent that provides online shoppers with personalized product rankings generated through the application of reinforcement learning to product/merchant information and consumer behavior/preferences. Garfinkel et al. [35] and Garfinkel et al. [36] developed a recommendation system that embeds an integer-programming model allowing users to choose the best products while taking into account cost savings through a bundling of products. Lim et al. [9] proposed a rule-based comparison-shopping framework using the eXtensible Rule Markup Language architecture, which computes the exact personalized delivery cost to find the optimal merchant.

## Intelligent Price Comparisons for Online Shoppers

Existing studies are limited in that they generally assume that shoppers are experts whose search strategies are direct, that is, the shoppers have clear product knowledge or preferences. In contrast, this study focuses on novice shoppers who have no clear and sufficient prior knowledge of the target product category during online shopping, and are often anonymous PCS users.

Moreover, uncertainties in an online shopping environment should be dealt with in order to provide novice shoppers with comprehensive purchase-decision support. There are two types of uncertainties for novice shoppers. First, their objectives inherently tend to be vague in that they may not have decided on the manufacturer, seller, or acceptable price of the product they are considering. Second, many PCSs use prices quoted by the merchants, which contain price dispersions, errors, and click baits. Such uncertainties, noise, and fuzziness have seldom, if ever, been considered in the context of online shopping; however, PCSs should become more robust to such factors.

To address these two issues, this study proposes an intelligent product search system that is developed by refining and extending the fuzzy-semantic information management system [5]. The proposed system extracts the linguistic semantics from the product price dispersion on the Web, and produces a personalized product list for individual online shoppers. In doing so, the system uses a novel semantic procedure based on fuzzy logic and data mining, and is robust to the uncertainties, noise, and fuzziness of online shopping environments. Consequently, it is expected that the proposed product search system enables novice shoppers to make purchase decisions in a more convenient and intelligent manner.

### 3.1 Decision-making process for a conventional price comparison site

Modern PCSs provide users with a vast amount of information on a broad range of products, including appliances, computers, clothing, and cosmetics. Therefore, an individual user first selects a product category they are interested in, and the PCS then displays a list of products belonging to the selected category. Because online shoppers tend to be price sensitive, this list often contains the lowest prices for each product. This is the situation with which PCS visitors are commonly faced.

**Figure 1:**
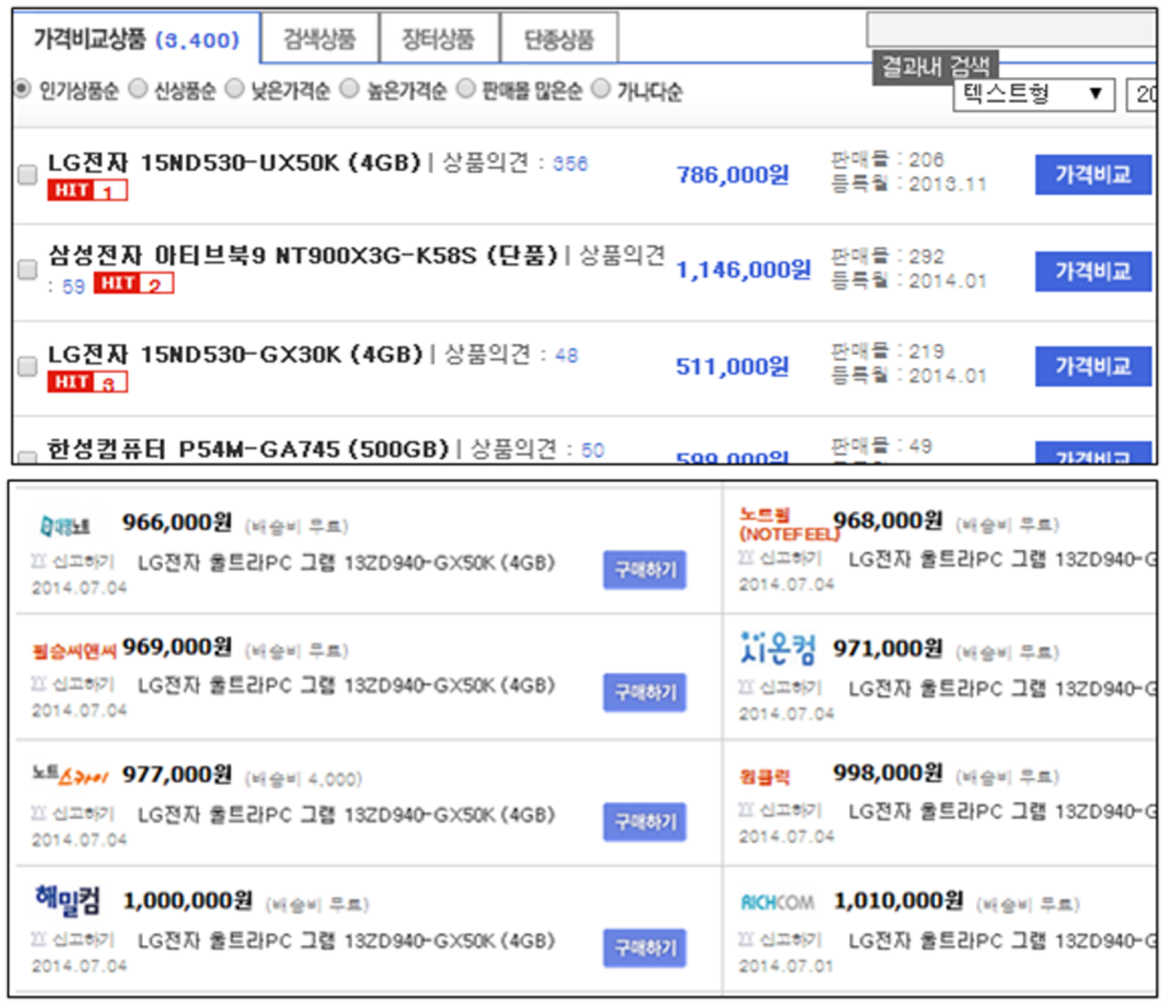
A conventional PCS: Products in the selected category (upper) and sellers/prices of the selected product (lower).

However, because a number of sellers charge different prices for identical products, users need to select a specific product from the product list to check the list of sellers and their prices. For example, Figure 1 shows a popular PCS in Korea. The upper panel in the figure lists products belonging to the category of “laptop computers,” and the lower panel lists the prices and sellers of a specific product.

**Figure 2:**
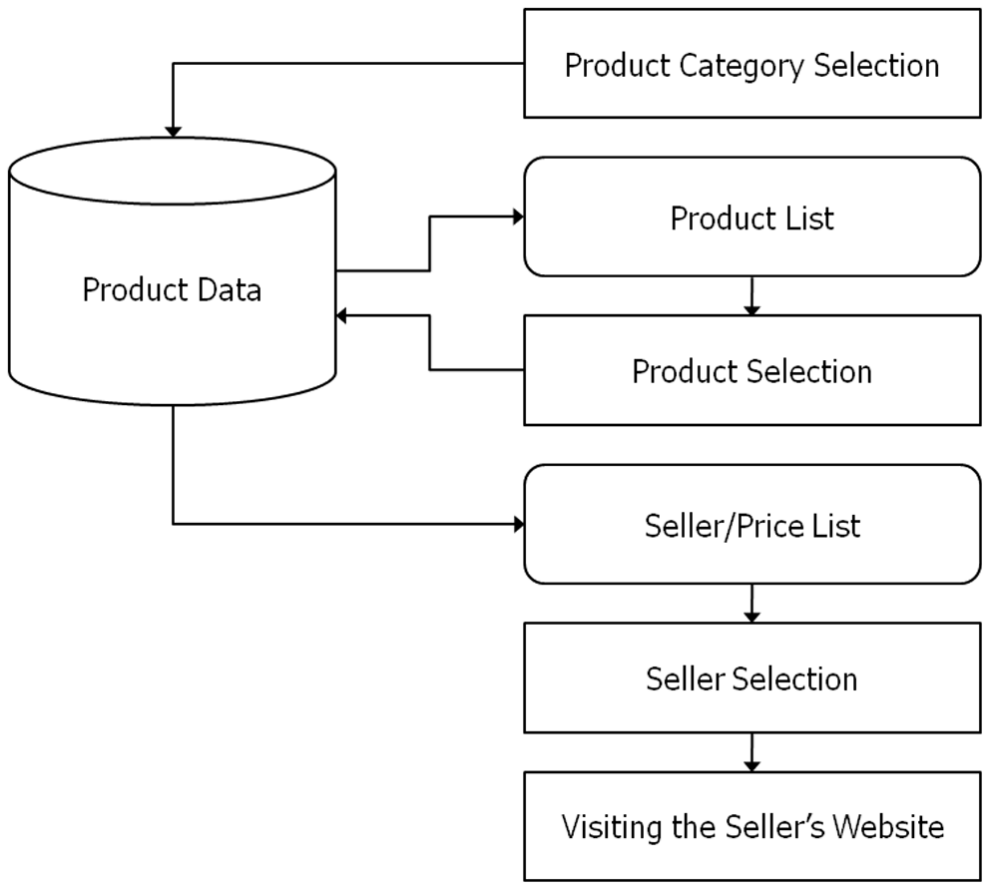
Decision-making process for the existing price comparison sites.

Considering a list of sellers offering a selected product, the user identifies the seller offering the best deal and can click on the hyperlink to that seller's Web page, where the user can obtain more information on the seller's offerings before making an actual purchase. Note that the role of a PCS differs from that of an online shopping mall in that individual users cannot make purchases directly on a PCS. That is, a PCS acts as an intermediary between individual online shoppers and sellers, and the main benefit of visiting such sites is that individual users can obtain information required for making a purchasing decision. Figure 2 summarizes the purchase decision-making process for existing PCSs.

However, users may have difficulty in making a purchasing decision using a PCS because of the massive amount of information provided by such sites. Although PCSs typically allow users to filter or sort the product and seller lists, as shown in Figure 1, for an efficient search, this problem still remains for the following two reasons:

(a) An individual user can be a novice with respect to the product that they wish to buy. That is, the user may have little prior knowledge regarding the target product category. In this case, it may be difficult for a novice shopper to efficiently filter through a list provided by a PCS and search for a desired product.

(b) User searches are often not direct in that a user may want to buy a product within a general product category despite not having decided on a specific product. Although users may browse and investigate the product and seller lists in an ad-hoc manner, this can be a time-consuming task because of the large amount of information provided by a modern PCS.

In this study, a novice online shopper is defined as a user satisfying both (a) and (b) above. In addition, it is clear that prices represent one of the most important factors in purchasing decisions, and that online shoppers tend to initiate the purchase decision-making process by establishing an approximate budget for their purchase items. For example, a novice shopper may intend to buy “a laptop computer for about $500” or “a laptop computer for $500 to $600.” In this case, the shopper who may lack sufficient domain knowledge regarding the product category is likely to be overwhelmed by the massive amount of information provided by the product and seller lists. Thus, they may have difficulty using an existing PCS and buying a product within a pre-determined product category.

Nevertheless, PCSs should be able to support online shoppers in an effective manner if they can appropriately facilitate the processing of the collected information. In this regard, focusing on novice shoppers interested in buying products under rough budget constraints, this study devises a novel framework under which PCSs can provide their users with intelligent support. Consequently, the following issues should be addressed:

(1) PCSs should provide novice shoppers with appropriate domain knowledge regarding the target product categories. For example, PCSs can determine whether a user's budget is more suitable for lower- or higher-priced products. Identifying the features of a product that provide a good fit based on the novice shopper's budget should help the shopper better understand the target product category and adjust their initial budget based on such features and their specific needs.

(2) PCSs should identify products that can be recommended to a novice shopper under the target product category. The novice shopper can then focus on the recommended products, which reduces their burden in terms of having to investigating a large amount of information.

In achieving objectives (1) and (2) above, the fuzziness of modern online shopping environments must be considered. A set of products that catch an individual user's interest are considered a fuzzy set, and not a crisp one. For example, consider a novice shopper wanting to buy a laptop computer within the price range of $500 to $600. Listing those products whose prices fall between $500 and $600 is a relatively easy task, but the user may also be interested in a laptop computer whose price falls within a different range. Similarly, a laptop computer that is $480 may be acceptable to a certain extent, although it may be less preferable than computers priced between $500 and $600.

Here, another case of fuzziness lies in the price of a given product. PCSs generally offer seller and price lists even for a single product. That is, the prices of a particular product on the Web may vary widely, and products are usually filtered and sorted according to their price. For most PCSs, the lowest price is generally used as the representative price of a given product. However, the lowest price of a given product is sometimes of little use for the following reasons: First, an exceptionally low price may be a mistake. Second, such prices tend to include products with limited specifications. Finally, such prices are generally achieved through special promotional campaigns. In this context, to enhance the relevance and understandability of the retrieved information, PCSs should address the limitations of using a crisp price in an appropriate manner.

### 3.2 Decision-making process using linguistic prices and linguistic product clusters

**Figure 3:**
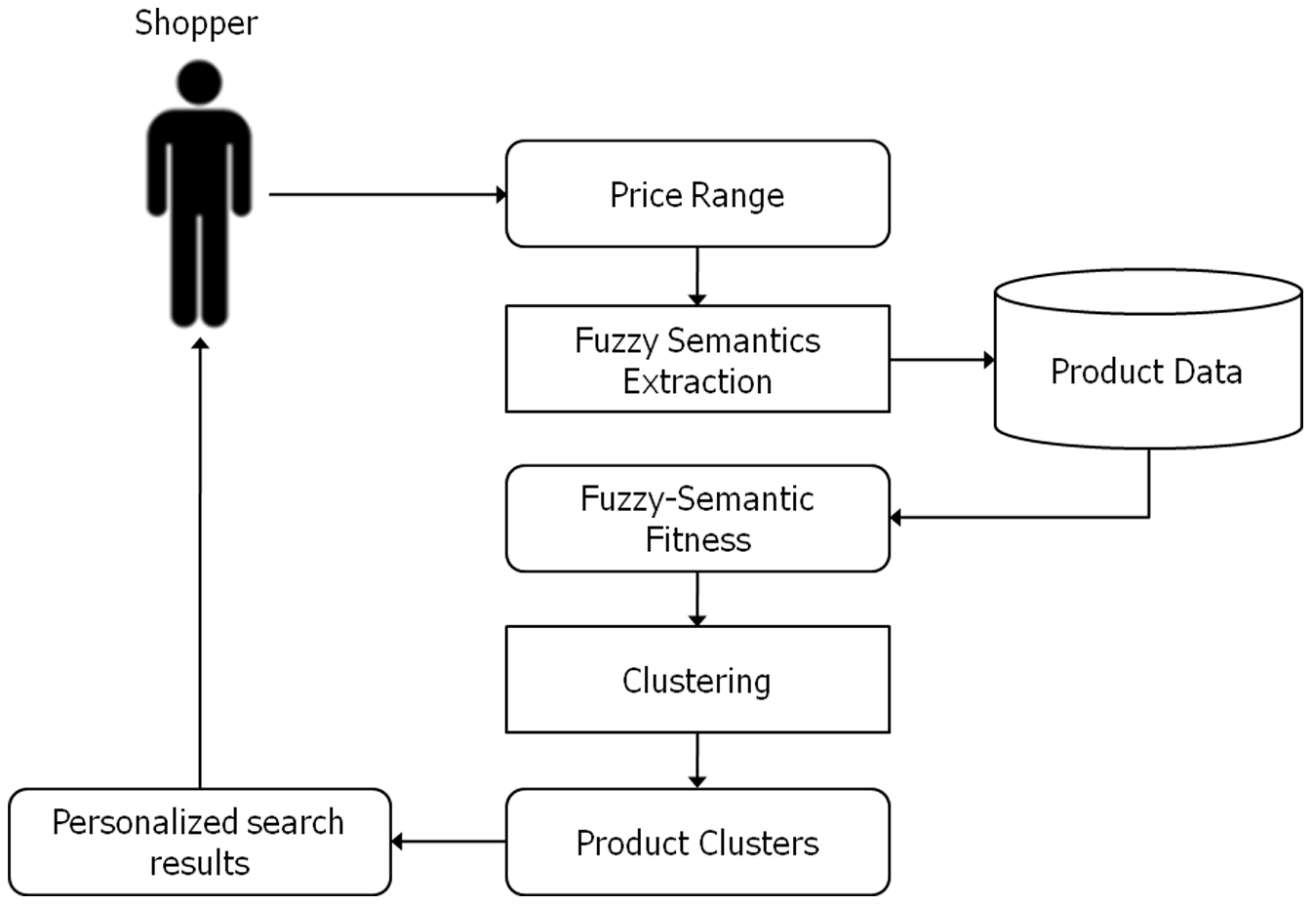
Overall framework of the intelligent product search system for online shoppers.

An intelligent product search system is devised to enhance the usability and relevance of PCSs for online shoppers (refer to Figure 3). The proposed system assumes that an individual user has already determined the target product category and has a rough budget. Therefore, the novice shopper first selects a product category based on the procedure for the particular PCS, and then sets a price range corresponding to their rough budget.

The intelligent product search system then extracts the fuzzy semantics of each product within the product category to produce the fuzzy-semantic fitness, which is a row vector containing a product's membership grade for linguistic price labels such as “cheap,” “good fit,” and “expensive,” to determine whether the product fits the price range set by the user. Next, the system clusters the products based on the fuzzy-semantic fitness vectors to obtain linguistic product clusters consisting of products with similar membership grades.

The proposed system uses these product clusters and the fuzzy-semantic fitness of each product to construct a personalized search result that provides the shopper with a better understanding of the target product category and that facilitates their purchasing decision.

#### 3.2.1 Linguistic prices based on fuzzy semantics

Fuzzy logic is a popular heuristic technique used for reasoning regarding the uncertainty inherent to words with ambiguous meanings. For an explanation of the fuzzy semantic extraction phase, consider an online shopper who wants to buy a laptop computer for $500 to $600. Existing PCSs can provide a list of laptop computers within this price range, but omit those computers that are priced below $500 or above $600, even though such computers may be attractive to the user. In this context, a set of products that fit the price range set by the user should be a fuzzy set.

Let *x* denote the price of a given product and the interval [*P*_min_, *P*_max_] denote the user-defined price range. If *x*<<*P*_min_, then the product is not recommended because its price is too low to satisfy the user. Similarly, if *x*>>*P*_max_, then the product is not affordable. Suppose that three linguistic values, *cheap* (*L*_1_), *good fit* (*L*_2_), and *expensive* (*L*_3_), can be assigned to a given product. Each linguistic value has an associated fuzzy set that indicates the degree of membership a specific price has within the set representing the associated linguistic value. For example, the fuzzy set associated with *cheap* maps each numeric price to a value between zero and 1. The output of the mapping indicates the probability that each price is a member of the *cheap* fuzzy set. Similar statements hold true for the fuzzy sets associated with *good fit* and *expensive*.

**Figure 4:**
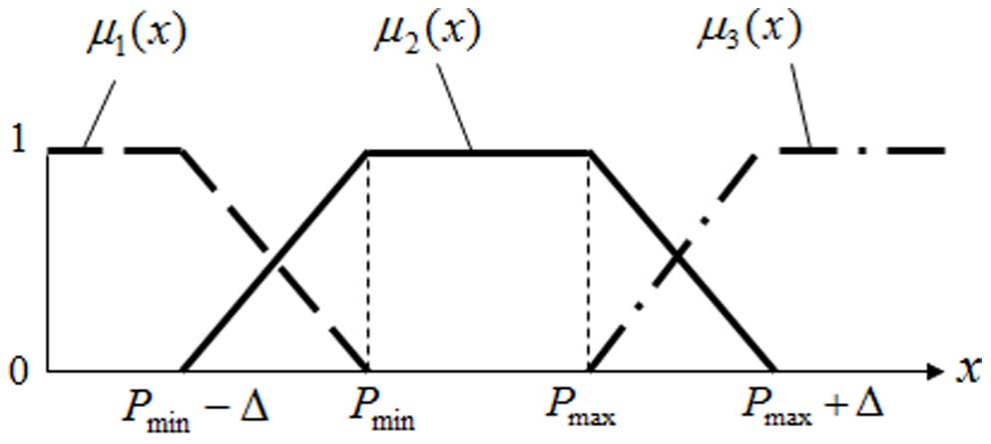
Three membership functions for price during fuzzy semantics extraction.

Figure 4 shows three membership functions (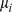) and their fuzzy sets for the linguistic value, *L_i_*: *cheap*, *good fit*, and *expensive*. It is clear that a finite fuzzy set for *cheap* cannot represent all possible numeric price values. A method for associating the probability of membership with a price not included in the definition of the fuzzy set is used to interpolate a membership score using values contained within the set.

The membership function for *L*_1_, *μ*_1_(*x*), is 1 if *x*≤*P*_min_–Δ, decreases if *x* is between *P*_min_–Δ and *P*_min_, and becomes zero if *x*>*P*_min_. Therefore, *μ*_1_(*x*) can be formulated as follows:
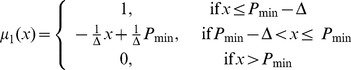
(3.1)


Similarly, we have
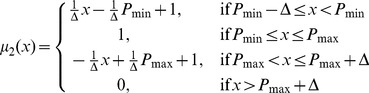
(3.2)

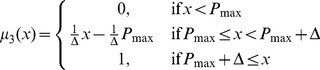
(3.3)


When *R* is the range of prices of a product within the target category, the “fuzzy-semantic fitness” is defined as a row vector, 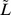 =  [*L*_1_ *L*_2_ *L*_3_]. If a single value is given to price *x*, the elements of 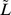 can be computed using equations (3.1) through (3.3). However, there are many sellers offering identical products at various prices under modern online shopping environments. In this context, a method for obtaining fuzzy semantic fitness 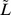 is developed as follows:

(1) Divide *R* into *n* intervals of the same length *l* ( = *R*/*n*) such that the prices are divided into *n* classes [*LP*_min_, *LP*_min_+*l*), [*LP*_min_+*l*, *LP*_min_+2*l*), …, [*LP*_min_+(*n*–2)*l*, *LP*_min_+(*n*–1)*l*), [*LP*_min_+(*n*–1)*l*, *LP*_min_+*nl*], where *LP*_min_ denotes the lowest price of a product in the target category.

(2) For a given product, a row vector 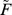 =  [*f*_1_ *f*_2_ … *f*_n_] represents the prices of a product, where *f_i_* is the relative frequency of those prices classified as class *i* (*i*≤*n*).

(3) However, users may still have difficulty in making a purchase decision based on the price vector. Buyers actually want to know whether a product is cheap, a good fit, or expensive. Therefore, the price vector needs to be mapped into the fuzzy semantic fitness vector, 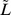 =  [*L*_1_ *L*_2_ *L*_3_], which represents three linguistic values, *L*_1_ (*cheap*), *L*_2_ (*good fit*), and *L*_3_ (*expensive*). This is achieved by making fuzzy inferences, 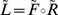, in which 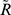 is a fuzzy relation, i.e., an *n*×*m* matrix, where *n* is the number of price intervals and *m* is the number of elements in 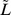 (*m* is 3 in this study):
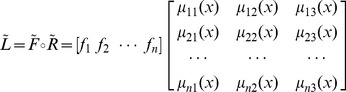
(3.4)where *μ_ij_*(*x*) is the membership grade in which price *x* in class *i* corresponds to linguistic value *L_j_*. *L_j_* can be computed by using (*f*_1_ ∧ *μ*_1*j*_(*x*)) ∨ (*f*_2_ ∧ *μ*_2*j*_(*x*)) ∨ … ∨ (*f_n_* ∧ *μ_nj_*(*x*)), where the operators ∨ and ∧ denote the *supremum* and *infimum* operations of the fuzzy set, respectively. The *supremum* operator outputs the maximum degree of membership, and the *infimum* operator outputs the minimum operands [14]. That is, *f*_1_ ∧ *μ*_1*j*_(*x*) = min(*f*_1_, *μ*_1*j*_(*x*)), and *f*_1_ ∨ *μ*_1*j*_(*x*) = max(*f*_1_, *μ*_1*j*_(*x*)).

For a clearer explanation, assume that *R* is 500,000 and *LP*_min_ is 100,000, and that there are five price intervals (*n* = 5). The price classes are then [100,000, 200,000), [200,000, 300,000), [300,000, 400,000), [400,000, 500,000), [500,000, 600,000]. Furthermore, price vector 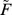 =  [*f*_1_ *f*_2_ *f*_3_ *f*_4_ *f*_5_] = [0.23 0.51 0.21 0.05 0.00] indicates that the product is low or medium priced because low-price intervals have high membership values.

Price vector 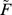 =  [0.23 0.51 0.21 0.05 0.00] can be mapped into 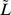 =  [*L*_1_ *L*_2_ *L*_3_] = [0.50 0.30 0.15] using the following fuzzy relation 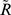:
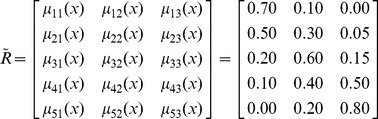
(3.5)where *μ*_11_(*x*) is 0.70, representing the membership grade in which price *x* in class [100,000, 200,000) corresponds to the linguistic value *cheap*. The value of *μ_ij_*(*x*) can be computed by using the following expression:
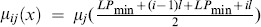
(3.6)where 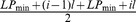 is the middle value of the interval. Therefore, *μ*_11_(*x*), *μ*_12_(*x*), and *μ*_13_(*x*) are calculated as follows:



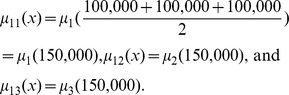



#### 3.2.2 Linguistic product clusters

Because there can be many products within the target category, the computed fuzzy-semantic fitness vector (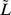) is further processed by using the proposed intelligent product search system to generate personalized search results for online shoppers. The intelligent product search system applies the *k*-means clustering algorithm to fuzzy-semantic fitness vectors, and groups products into several clusters. The proposed system then assigns appropriate linguistic labels to these product clusters, thereby enabling online shoppers to obtain a quick insight into the target product category and select the clusters of interest. If a cluster is selected, then the products in that cluster are listed, and an online shopper can find appropriate products in a convenient manner.

**Figure 5:**
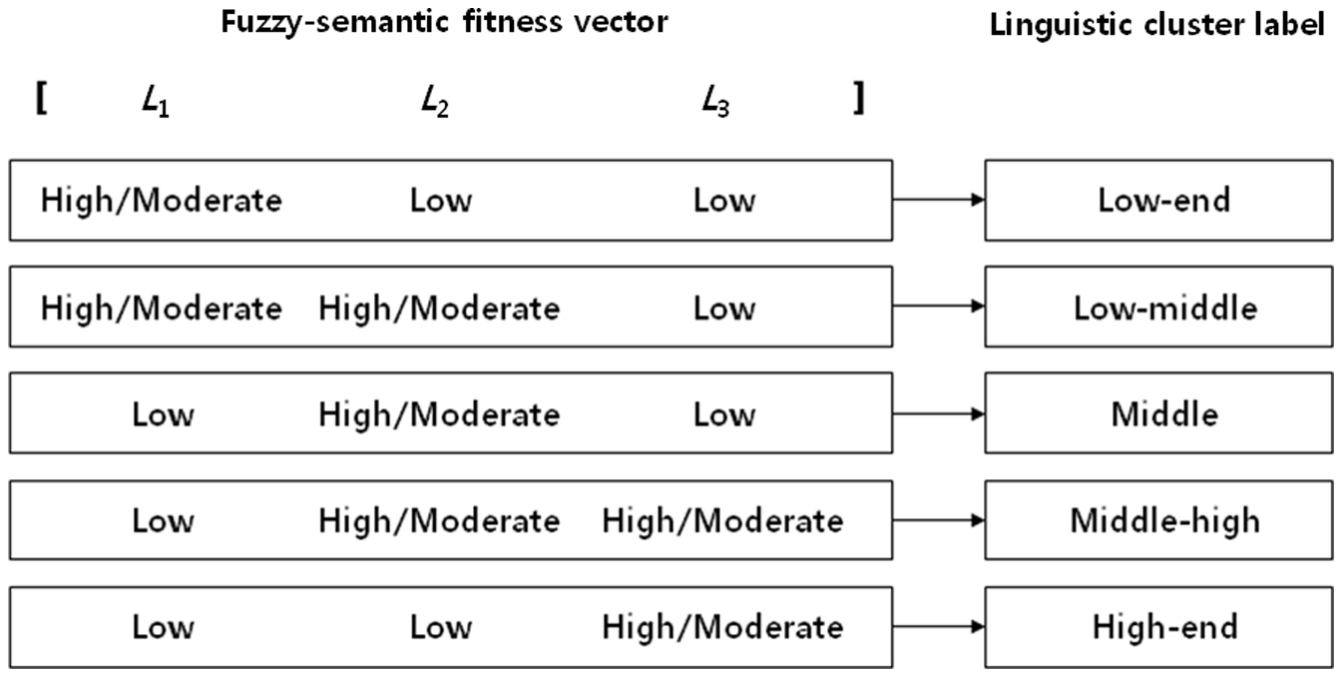
Five types of well-organized product cluster labels.

Although product prices may vary on the Web, they are generally distributed within a specific range. This suggests that the centroid of a well-organized product cluster corresponds to one of the five types shown in Figure 5. Because a product cluster should consist of products with similar fuzzy-semantic fitness vectors, 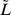 =  [*L*_1_ *L*_2_ *L*_3_], the centroid of a well-organized product cluster consists of one moderate-to-high value and two low values, or two moderate-to-high values for two consecutive elements of the fuzzy-semantic fitness vector and one low value for the remaining element. Note that one of linguistic cluster labels (from ‘Low-end’ to ‘High-end’) is assigned to each type of centroid, and this is a simple method for characterizing product clusters based on fuzzy-semantic fitness vectors.

After the clustering analysis is complete, the intelligent product search system provides the user with linguistic product clusters and their centroids, thereby enabling the user to gain knowledge regarding the target product category and select product clusters of interest in a convenient manner. When the user selects a product cluster, the proposed system lists those products belonging to the cluster by sorting the products based on the Euclidean distance (*Dist*) between each product's fuzzy-semantic fitness vector and the centroid of the cluster because the characteristics of the centroid induce the user to select that product cluster. This procedure allows individual online shoppers with a rough budget to find appropriate products from a sorted list.

## Experimental Results

### 4.1 Application to a price comparison site

To illustrate how the proposed system works, the procedure shown in Figure 3 was applied to one of the most popular PCSs in Korea. For this experiment, it was assumed that an online shopper was considering “ultra-thin laptop computers” as the target product category, and that the PCS they used gathered information on 95 products. Table 1 shows a sample of the collected products (to see the data used in this study, refer to Data S1 [37]). Here, KRW indicates South Korea's currency, the Korean won (as of January 2014, 1,070 KRW is equivalent to $1 USD). As shown in the table, there are many products whose prices vary considerably.

**Table 1:** A sample list of ultra-thin laptop computers (unit: KRW).

Product no.	# of sellers	Lowest price	Highest price	Price range
1	50	1,026,000	1,349,000	323,000
2	44	498,900	633,000	134,100
3	48	833,460	940,000	106,540
4	59	820,030	1,225,350	405,320
5	67	908,180	1,499,640	591,460
6	43	852,580	988,800	136,220
7	44	988,820	1,958,720	969,900
8	48	753,620	944,620	191,000
9	51	802,170	1,045,800	243,630
10	51	669,260	797,000	127,740
…	…	…	…	…
95	5	1,034,110	1,255,190	221,080

The PCS currently provides its users with a summarized list, as shown in Table 2. The products on the list are initially sorted based on their popularity, but can also be sorted according to price, the number of sellers, the release date, or in alphabetical order of the product names based on the user's preference. Note that the table shows the lowest prices and cannot provide users with a deep insight into the products.

**Table 2:** Initial product list for the PCS (unit: KRW).

Product no.	Description	Manufacturer	# of sellers	Lowest price
1	Xnote P210-GE30K	LG	50	1,026,000
2	ThinkPad Edge 0328-RU5	Lenovo	44	498,900
3	SENS NT-X280-PA55S	Samsung	48	833,460
4	Xnote P210-GE2PK	LG	59	820,030
5	TouchSmart TM2-2107fx	HP	67	908,180
6	SENS NT-X280-PA65S	Samsung	43	852,580
7	Xnote P210-GEP3K	LG	44	988,820
8	SENS NT-X180-BARBIE	Samsung	48	753,620
9	Xnote P210-GE1AK	LG	51	802,170
10	SENS NT-X180-JA33S	Samsung	51	669,260
…	…	…	…	…
95	SENS NT-X360-AA31R	Samsung	5	1,034,110

If an individual user selects a specific product (e.g., product 1) from the list in Table 2, then those sellers offering this product and their prices are also listed, as shown in Table 3. In addition, clicking on the name of a seller allows the user to visit the seller's online shopping mall, where they can actually purchase the product. Conventional PCSs typically allow users to filter products according to their prices, and users naturally focus on those products that fall within a specific price range to reduce the amount of retrieved information. However, users may be overwhelmed by the massive amount of information shown in the lists in Tables 2 and 3, and thus may have difficulty in making a purchasing decision through a conventional PCS.

**Table 3:** Sellers and prices for product 1 (unit: KRW).

	Seller	Price
1	G-market	1,026,000
2	Auction	1,026,060
3	11st	1,026,790
…	…	…
50	11st	1,349,000

### 4.2 Linguistic price comparisons and personalization

Now, let us consider an individual user who wants to buy an ultra-thin laptop computer within the price range of 800,000 to 1,000,000 KRW. Although conventional PCSs generally provide a list of products with the lowest prices within the specified price range, several problems remain. For example, product 1 in Table 1 is excluded from the filtered product list, although its lowest price is very close to 1,000,000 KRW and may be attractive to the user. Similarly, product 10, whose highest price is very close to 800,000 KRW, is also excluded. Therefore, the user cannot identify these products through the filtered product list. In contrast, although products 5 and 7 are considered expensive because their highest prices far exceed 1,000,000 KRW, they are included in the filtered product list. These problems are addressed by employing the proposed system to provide the user with relevant information for a purchasing decision.

Here, *LP*_min_, the lowest product price in the target category, was 378,900 KRW, and *R*, the range of product prices for this category, was 1,039,870 KRW. The proposed system then calculated *l* = 50,000 (≈*R/n* = 1,039,870/20) for an *n* of 20 (a sufficiently large *n* was used while taking into account computational convenience), and represented the price vectors of the products as shown in Table 4, where Δ = *R*/10. The fuzzy relation 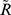 and price vector 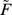 were employed to obtain the fuzzy-semantic fitness vector 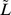 shown in Table 5. The linguistic product clusters were then obtained in Table 6 by applying the *k*-means clustering algorithm to 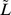.

**Table 4:** Price vectors of products presented by the relative frequency.

Product no.	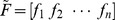 (*n* = 20)
1	[0.00 0.00 0.00 0.00 0.00 0.00 0.00 0.00 0.00 0.00 0.00 0.00 0.32 0.36 0.18 0.10 0.00 0.00 0.04 0.00]
2	[0.00 0.07 0.52 0.30 0.11 0.00 0.00 0.00 0.00 0.00 0.00 0.00 0.00 0.00 0.00 0.00 0.00 0.00 0.00 0.00]
3	[0.00 0.00 0.00 0.00 0.00 0.00 0.00 0.00 0.33 0.48 0.19 0.00 0.00 0.00 0.00 0.00 0.00 0.00 0.00 0.00]
4	[0.00 0.00 0.00 0.00 0.00 0.00 0.00 0.00 0.22 0.10 0.07 0.14 0.17 0.14 0.14 0.02 0.02 0.00 0.00 0.00]
5	[0.00 0.00 0.00 0.00 0.00 0.00 0.00 0.00 0.00 0.00 0.10 0.01 0.34 0.19 0.06 0.04 0.12 0.03 0.03 0.06]
6	[0.00 0.00 0.00 0.00 0.00 0.00 0.00 0.00 0.00 0.67 0.23 0.09 0.00 0.00 0.00 0.00 0.00 0.00 0.00 0.00]
7	[0.00 0.00 0.00 0.00 0.00 0.00 0.00 0.00 0.00 0.00 0.00 0.11 0.43 0.32 0.07 0.05 0.00 0.00 0.00 0.02]
8	[0.00 0.00 0.00 0.00 0.00 0.00 0.00 0.46 0.40 0.13 0.02 0.00 0.00 0.00 0.00 0.00 0.00 0.00 0.00 0.00]
9	[0.00 0.00 0.00 0.00 0.00 0.00 0.00 0.00 0.55 0.29 0.08 0.00 0.08 0.00 0.00 0.00 0.00 0.00 0.00 0.00]
10	[0.00 0.00 0.00 0.00 0.00 0.43 0.29 0.27 0.00 0.00 0.00 0.00 0.00 0.00 0.00 0.00 0.00 0.00 0.00 0.00]
…	…
95	[0.00 0.00 0.00 0.00 0.00 0.00 0.00 0.00 0.00 0.00 0.00 0.00 0.60 0.00 0.00 0.00 0.20 0.20 0.00 0.00]

**Table 5:** Fuzzy-semantic fitness vectors of products.

Product no.	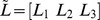
1	[0.00 0.32 0.36]
2	[0.52 0.00 0.00]
3	[0.00 0.48 0.00]
4	[0.00 0.22 0.17]
5	[0.00 0.34 0.25]
6	[0.00 0.67 0.00]
7	[0.00 0.43 0.32]
8	[0.25 0.46 0.00]
9	[0.00 0.55 0.08]
10	[0.43 0.27 0.00]
…	…
95	[0.00 0.60 0.25]

**Table 6:** Linguistic product clusters with centroid information.

Cluster	# of products	Centroid of the cluster			Linguistic label
		*L*_1_	*L*_2_	*L*_3_	
1	17	0.62	0.05	0.00	Low-end
2	16	0.33	0.33	0.01	Low-middle
3	6	0.04	0.93	0.03	Middle
4	14	0.02	0.59	0.10	Middle
5	16	0.00	0.40	0.17	Middle
6	21	0.00	0.27	0.25	Middle-high
7	5	0.00	0.02	0.65	High-end

The proposed system grouped products into seven clusters and assigned linguistic labels (from Low-end to High-end) to each cluster. In addition, the system sorted these clusters based on the labels. Therefore, a novice shopper can acquire knowledge quickly regarding the structure of product prices within the target category.

If an online shopper is interested in choosing a specific product cluster, they can select that cluster to see the products it contains. Because the shopper in the present example used a price range of 800,000 to 1,000,000 KRW for the product search, the shopper is interested in products labeled Low-middle. The shopper then selects cluster 2 in Table 6, and is provided with a list of products belonging to that cluster, as shown in Table 7. The products in cluster 2 are sorted in ascending order of *Dist*. The fuzzy-semantic fitness vectors of the products in the same cluster are similar to each other. Therefore, these products are expected to satisfy shoppers interested in products labeled Low-middle. Furthermore, product 10, which was excluded through conventional PCS filtering, is identified, as shown in Table 7.

**Table 7:** Products in cluster 2.

Product no.	# of sellers	Lowest price	Highest price	*L*_1_	*L*_2_	*L*_3_	*Dist*
82	3	793,800	973,800	0.25	0.33	0.00	0.08
76	9	767,340	990,000	0.25	0.33	0.00	0.08
69	16	690,000	916,340	0.25	0.31	0.00	0.08
29	43	765,520	1,050,000	0.25	0.35	0.05	0.09
50	13	784,000	975,000	0.23	0.31	0.00	0.10
53	21	767,340	1,010,000	0.25	0.38	0.05	0.10
10	51	669,260	797,000	0.43	0.27	0.00	0.12
…	…	…	…	…	…	…	…

If an online shopper wanting to find products labeled Middle-high selects cluster 6 in Table 6, they are then provided with the list of products shown in Table 8. Similarly, it can be seen that product 1, which was excluded by the conventional PCS, is included in the product list, although its priority is relatively low depending on the distance from the centroid.

**Table 8:** Products included in cluster 6.

Product no.	# of sellers	Lowest price	Highest price	*L*_1_	*L*_2_	*L*_3_	*Dist*
15	60	943,610	1,224,000	0.00	0.28	0.25	0.01
67	12	935,010	1,400,000	0.00	0.25	0.25	0.02
16	36	819,580	1,492,500	0.00	0.25	0.22	0.04
21	32	881,980	1,112,000	0.00	0.31	0.25	0.04
84	9	869,790	1,270,000	0.00	0.22	0.22	0.06
…		…	…	…	…	…	…
1	50	1,026,000	1,349,000	0.00	0.32	0.36	0.12
…	…	…	…	…	…	…	…

As demonstrated above, the intelligent product search system proposed in this study can provide online shoppers with a quick insight into the target product category and knowledge about the distribution of prices within that category, and help them identify the appropriate products. In addition, this system does not exclude products even if they are outside the user-defined price range. In this way, online shoppers can make better purchasing decisions even when they do not have sufficient prior knowledge regarding the target product categories.

### 4.3 Prototype of the intelligent product search system

A prototype system was developed using a common Web programming language, Java Server Page, and MS-SQL Server was used as the data repository. Moreover, the Google Charts library was deployed to provide the users with a visual aid.

**Figure 6:**
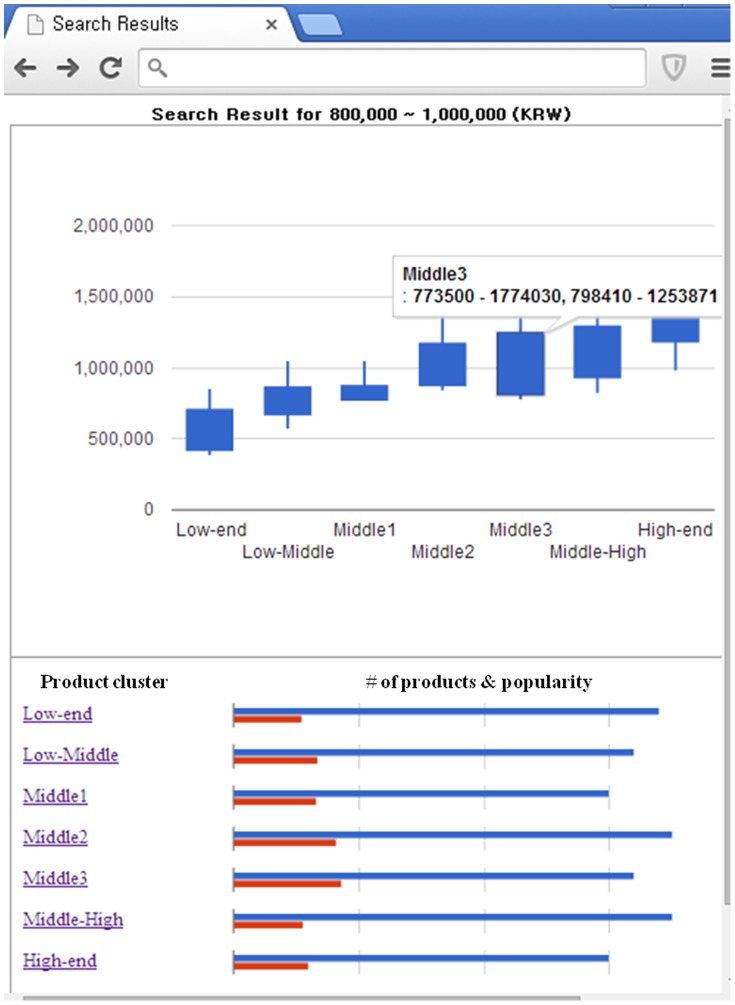
A cluster summarization page for the specified product category and price range.

For the price range specified by the user, the prototype system first generated a cluster summarization page. Figure 6 shows a summarization of the product clustering in the ultra-thin laptop computer category for a price range of 800,000 to 1,000,000 KRW. In the upper part of this page, the price dispersion of each product cluster was represented through a candlestick chart. The top and bottom of a body were determined based on the minimal and maximal values of the average product price. The end points of a vertical line were specified based on the minimal and maximal prices of the corresponding cluster. Moreover, the details of the price dispersion of a product cluster can be checked by clicking on the candlestick chart. The figure also shows the details of the *Middle 3* cluster, for example.

In the lower part of the cluster summarization page, the number of items and average popularity of each product cluster were visualized through a bar chart. Therefore, the users can conveniently obtain insight into the products included in this product category and narrow the scope of their search.

After choosing a product cluster to be further investigated, the user can move to a product list page by clicking on the product cluster name in the lower-left corner of the cluster summarization page. As shown in Tables 7 and 8, the products included in the selected product cluster were first sorted based on the distance to the cluster centroid, and the users may sort them using other criteria such as the product name, lowest price, highest price, and number of sellers.

**Figure 7:**
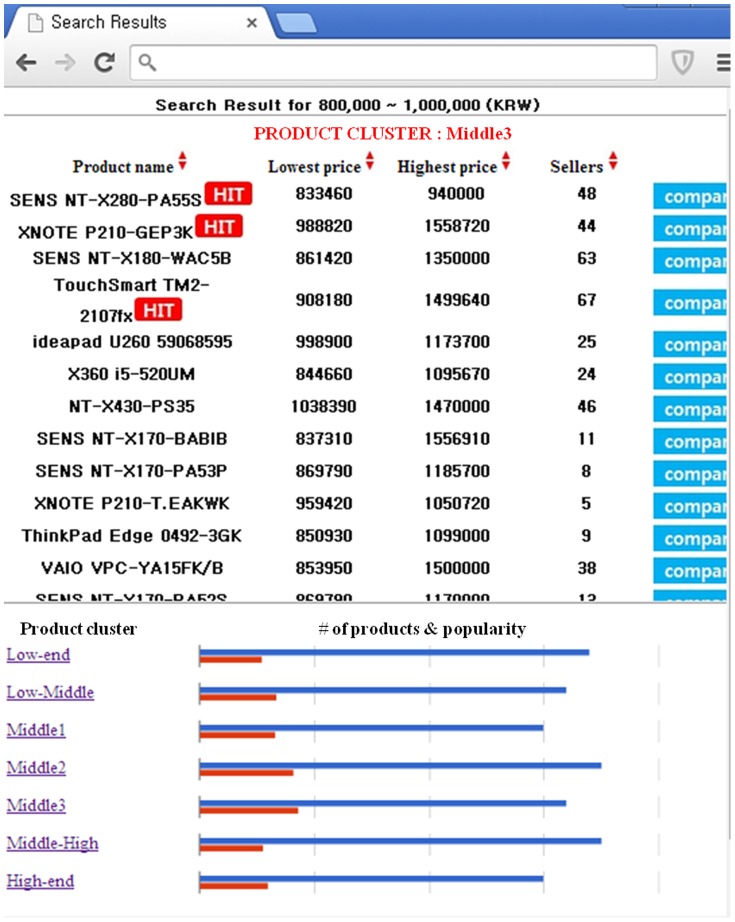
A product list page for the selected cluster.

Figure 7 shows the product list page for the product cluster *Middle 3*. The first product, “SENS NT-X280-PA55S,” can be labeled as *Middle* considering its price range. On the contrary, the price range of the 7th item, “NT-X430-PS35,” does not overlap with the user-specified price range. Indeed, it is reasonable for the product cluster to include this product because most of its prices are very close to 1,000,000 KRW; however, such products are not considered in a traditional PCS. In this way, the proposed intelligent product search system enables users to find attractive products in more convenient manner, and provides online merchants with the opportunity for potential sales.

Because, in a conventional PCS, products with different price labels are listed on a single page, where hundreds of products are occasionally listed, interpreting such an enormous amount of information is very inefficient, and users with only a rough budget may be discouraged to make a purchase. Therefore, it can be seen that the semantic procedures, product clusters, and visual aids are very useful for providing users with proper guidance, and the proposed system will be very helpful in supporting novice shoppers.

## Conclusions and Future Research

Information-intensive Web sites provide a wide access to a diverse range of information sources. However, the browsing behaviors of many users are not directed in that users do not focus on locating specific targets and often experience problems of information overload and uncertainty. In particular, novice users have a considerable difficulty in making decisions based on information provided by such Web sites. Therefore, providing online users with useful information is a major challenge facing future Web environments.

As an example of information-intensive Web sites that can accommodate the needs of online users, this study considered a conventional PCS that has faced a user's vague search objectives and the uncertainty surrounding online shopping environments. To address these problems, an intelligent product search system was developed to provide online shoppers with quick insight into a product category and help them identify appropriate products in a more convenient manner.

The proposed system extracted the linguistic semantics hidden in product price dispersion using fuzzy logic, and employed the *k*-means clustering algorithm to generate linguistic product clusters for personalized results. In this regard, the characteristics of well-organized linguistic product clusters were outlined and used for a clustering analysis.

Once the price range is specified, the proposed system first displays a summarization page of the product clusters. This page shows the price labels, price dispersions, numbers of included products, and average popularity of the product clusters, and the users can conveniently choose a cluster suitable for their needs. After a product cluster is selected, a product list page is generated by taking the user-specified price range and user preferences into account; in addition, visual aids also help the users understand the search results. Consequently, online shoppers can find suitable products in a more effective way.

Although the proposed system addresses important issues inherent to a conventional PCS, there are still several research topics to further investigate. First, the intelligent product search system is limited in that it only considers product prices and user-defined price ranges. In this regard, future research should consider other factors that can be useful for generating personalized product recommendations, such as the user's preferred vendors, the product specifications, and promotional campaigns. Moreover, the advantages of the proposed system should be empirically validated through future research, which applies the proposed system to a wide range of product categories.

## Supporting Information

Data S1 **The information of “ultra-thin laptop computers” collected by a price comparison site.**
(XLS)
